# Multifunctional Electrospun Nanofibers for Biosensing and Biomedical Engineering Applications

**DOI:** 10.3390/bios14010013

**Published:** 2023-12-25

**Authors:** Zhou Chen, Mengdi Guan, Yi Bian, Xichen Yin

**Affiliations:** School of Mechanical and Power Engineering, Nanjing Tech University, Nanjing 211800, China; 202161107030@njtech.edu.cn (M.G.); 202221106001@njtech.edu.cn (Y.B.); 202021106002@njtech.edu.cn (X.Y.)

**Keywords:** electrospinning, nanofibers, biosensor, biomedicine

## Abstract

Nanotechnology is experiencing unprecedented developments, leading to the advancement of functional nanomaterials. The properties that stand out include remarkable porosity, high-specific surface area, excellent loading capacity, easy modification, and low cost make electrospun nanofibers. In the biomedical field, especially in biosensors, they exhibit amazing potential. This review introduces the principle of electrospinning, describes several structures and biomaterials of electrospun nanofibers used for biomedicine, and summarizes the applications of this technology in biosensors and other biomedical applications. In addition, the technical challenges and limitations of electrospinning for biomedicine are discussed; however, more research work is needed to elucidate its full potential.

## 1. Introduction

As living standards improve, better health and rehab after injuries and illnesses are expected, making biomedical engineering ever more sophisticated and precise [1]. Biomedical materials have attracted great interest as a source of diagnostics, treatment, or replacement in biological organisms. The materials used for biomedical purposes need to satisfy several strict requirements: they must be biocompatible, avoiding any adverse reactions in the body; the materials must have bioactivity to trigger an effect on living cells, tissues, or organisms; and they should possess sufficient physical and mechanical properties to be processed easily [2,3]. Thus, direct contact and interaction with biological systems make the selection of biomedical materials a tremendous challenge.

**Figure 1:**
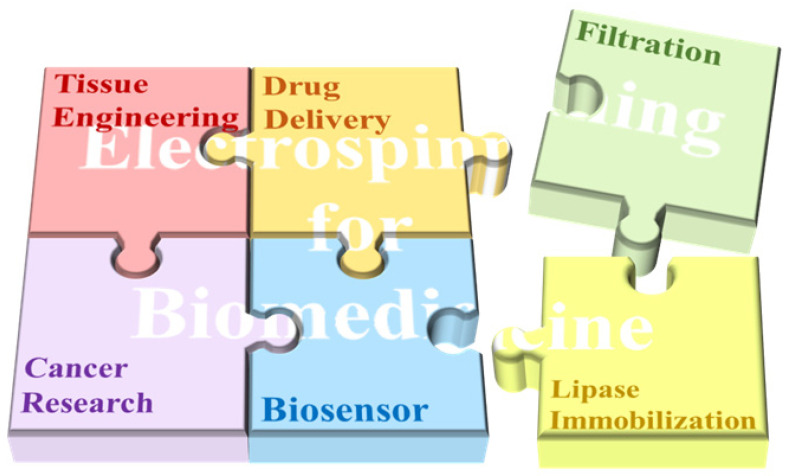
The major applications of electrospinning technology in biomedicine.

Nanomaterials with unique physical and chemical properties have become viable biomedical materials as a result of advances in nanotechnology and transdisciplinary research [4]. Among them, nanofibers have sparked a lot of interest in biomedical applications for their biomimetic property and ease of production [5,6]. Electrospinning is a straightforward technology forming continuous nanoscale fibers [7]. Electrospun nanofibers are characterized by their large porosity, superior specific surface area, easy modification, and low cost. Recently, as shown in Figure 1, they have been widely utilized in many areas of biomedicine such as tissue engineering, drug delivery, cancer research, biosensors, filtration, and lipase immobilization [8,9,10,11,12]. Nanofiber-based biosensors provide many benefits over traditional biosensors, including heightened responsiveness, increased sensitivity, broader detection capabilities, and cost effectiveness. This article reviews the operating principles of electrospinning, typical structures of electrospun nanofibers for biomedical usage, and the applications of electrospun nanotechnology for biosensors and other biomedical fields.

## 2. Overview of Electrospinning

### 2.1. General Working Principle of Electrospinning

**Figure 2:**
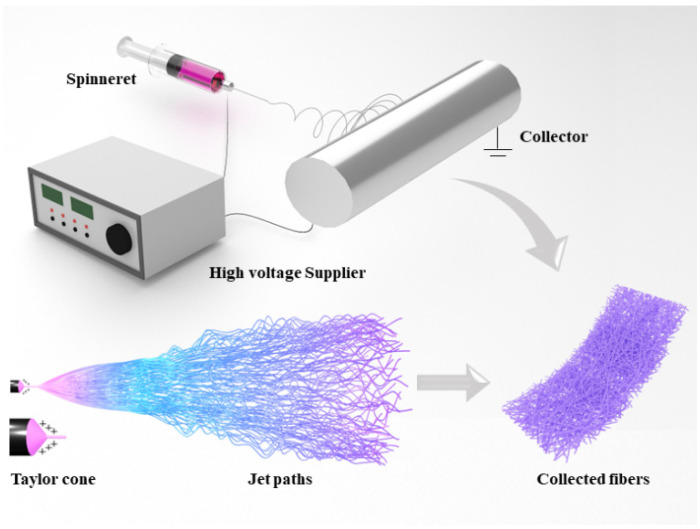
Schematic diagram of a typical electrospinning device.

Electrospinning fabricates nanofibers by applying electrostatic forces to uniaxially stretch a viscoelastic solution [13]. As shown in Figure 2, a typical electrospinning device consists of a high-voltage supplier, a spinneret, and a collector. When a high voltage is applied between the spinneret and collector, owing to the existence of electrostatic forces, the charged droplet at the top of the spinneret deforms into a typical conical-shaped droplet called a Taylor cone [14]. The electrostatic force makes the jet fly to the collector as it overcomes the surface tension of the droplet. The jet extends further and becomes finer under the influence of electrostatic forces, coagulates due to the evaporation of the solvent, and finally deposits as fibers on the collector, which is grounded [15].

Conventional methods for preparing nanofibers include drawing, stretching, chemical vapor deposition, self-assembly, and so on. Compared with those production approaches, there are several advantages of electrospinning: (1) convenience and effective cost; (2) easy integration of bioactive substance into fibers; and (3) almost no process heat, which is beneficial for sensitive compounds [16]. Electrospun fibers are very small in diameter and have a large specific surface area and a high porosity. Based on these merits, electrospinning exhibits potential applications in the field of biomedicine. 

### 2.2. Different Electrospinning Approaches

#### 2.2.1. Coaxial Electrospinning

**Figure 3:**
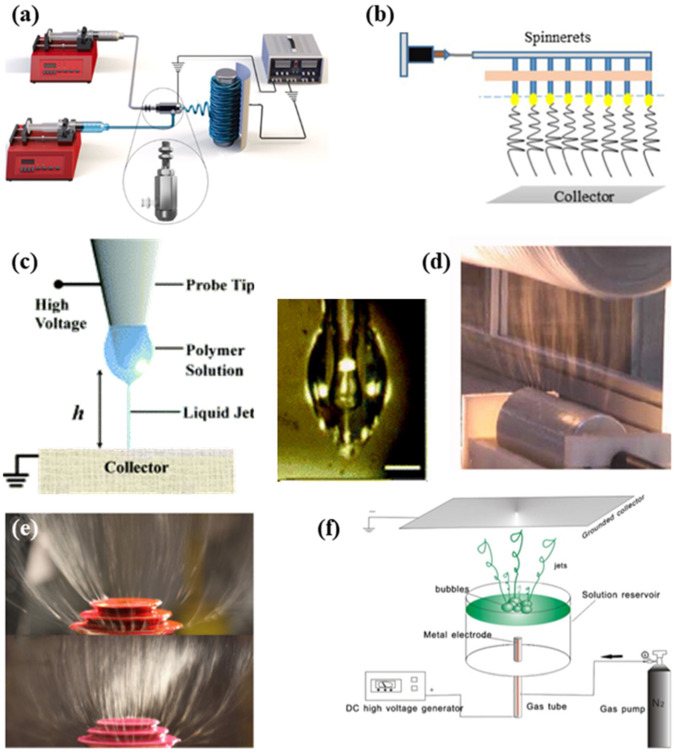
Schematic diagram of (**a**) coaxial electrospinning [20], (**b**) multi-needle electrospinning [21], (**c**) near-field electrospinning [22], (**d**) needleless electrospinning with rotary spinneret devices [23], (**e**) needleless electrospinning with a stationary spinneret device (stepped pyramid) [24], and (**f**) needleless electrospinning with a stationary spinneret device (bubble spinneret) [25].

The methodology of coaxial electrospinning was first proposed in 2002 and is now widely used [17]. The device of this electrospinning approach is shown in the diagram in Figure 3a. It can generate nanofibers with a core–shell structure. Compared with the conventional electrospinning device, coaxial electrospinning has some improvements in the spinneret part: the original single spinneret is replaced by a typical spinneret assembled by two coaxial capillaries. Each capillary provides the corresponding solution by different pumps, which effectively inhibits solution mixing. Under the action of high-pressure electrostatic force, a coaxial Taylor cone forms at the top of the coaxial spinneret, followed by a coaxial fluid jet [18]. Finally, the nanofibers with a core–shell structure are created on the collector. The application of core–shell nanofibers will be described in Section 3.2.1. If the core is removed, coaxial electrospinning can also yield hollow fibers that have large surface-to-volume ratios [19]. In addition, many precursor solutions that are considered “non-spinnable”, such as low molecular weight polymer solutions, can be spun by coaxial electrospinning [17]. They are used as cores, with spinnable solutions as shells, and the shells can be subsequently removed by post-treatment.

#### 2.2.2. Multi-Needle Electrospinning

The inability to achieve industrial mass production has been the problem faced by the traditional electrospinning process. Multi-needle electrospinning technology, which increases the number and density of needles to further develop the creation proficiency of nanofibers, is an approach for large-scale manufacturing of nanofibers (Figure 3b). Nanofibers made by this approach have better uniformity and adaptability than other electrospinning mass production mechanisms. Nevertheless, due to the increased number of needles, the distance between each needle tends to be short [19]. This results in interference of electric fields at the needle tips, resulting in instability of jets and producing fibers of relatively poor quality; hence, the calculation of the process parameters needs to be precise [26].

#### 2.2.3. Near-Field Electrospinning

Nanofibers generated by the conventional electrospinning technique are usually disordered, which in some ways limits their further application. In 2006, Sun et al. invented an electrospinning approach called near-field electrospinning, which has a shorter spinning distance as shown in Figure 3c. The deposition can be controlled during the near-field process [27]. Additionally, this electrospinning approach reduces the spinning voltage effectively. This method makes it possible to control the location of fiber deposition and produce aligned nanofibers, which broaden its access to biomedical fields such as tissue engineering and biosensors. He et al. used near-field electrospinning to produce composite scaffolds made of polycaprolactone (PCL) and hydroxyapatite (Hap). This study leads to a brand-new method for the production of 3D scaffolds used in tissue engineering. Currently, the major drawback of this approach is the limitation in mass production because of the poor quantity of the dipped solution.

#### 2.2.4. Needleless Electrospinning

Needleless electrospinning is another widely used industrial production approach for electrospinning. Conventional electrospinning devices applying needles as spinnerets are prone to clogging [28]. Needleless electrospinning can eliminate such phenomena. They can be divided into two kinds through the state of the spinneret: rotary spinneret electrospinning and stationary spinneret electrospinning [29]. The rotary spinneret devices frequently used are the cylinder, ball, disk, and wire. Figure 3 shows a cylindrical spinneret. Stationary spinneret electrospinning is a technology that relies on external forces to generate a Taylor cone. Figure 3e,f show the schematic diagram of common stationary spinneret devices with the stepped pyramid spinneret and bubble spinneret. However, because needleless electrospinning cannot control the jet orientation, jetting time, or jet size, its development encounters bottlenecks [30].

### 2.3. Electrospun Nanofibers

**Figure 4:**
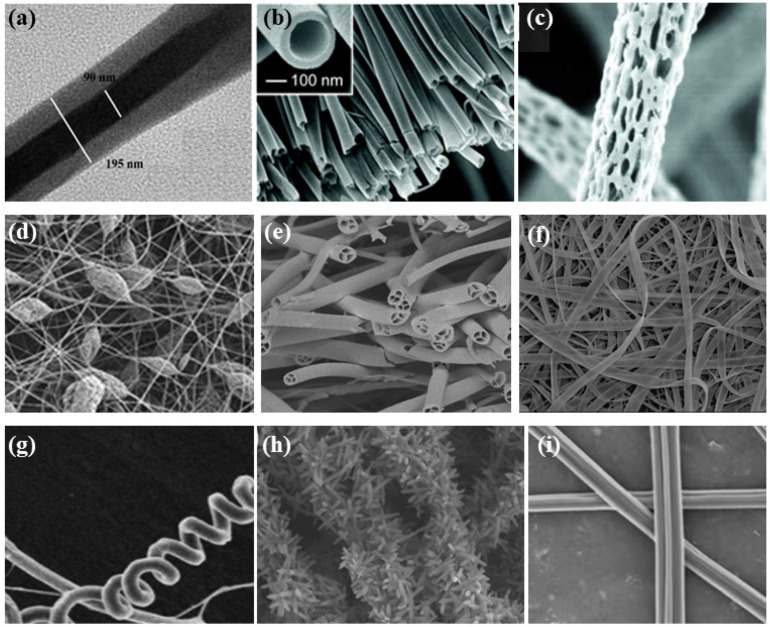
Different electrospun nanofibers structures: (**a**) core–shell fibers [41]; (**b**) hollow fibers [42]; (**c**) porous fibers [43]; (**d**) beaded fibers [44]; (**e**) multi-channel fibers [45]; (**f**) ribbon fibers [46]; (**g**) helical fibers [47]; (**h**) pine-needle-like fibers [48]; (**i**) side-by-side fibers [49].

Normal electrospun nanofibers are made from uniaxial electrospinning with s smooth surface and disorderly arrangement. Because such fibers tend to have a single function and gradually fail to meet people’s demands, much research is now focused on innovation of the fiber structures. Figure 4 shows different electrospun nanofibers structures.

The general way to obtain fibers with special structures is to adjust electrospinning parameters [31]. Several parameters, such as solution parameters, operational conditions, and ambient factors, exist in the process of electrospinning. The raw material of electrospun nanofibers can be natural or synthetic polymer solutions with different concentrations, viscosities, and volatilities [32]; During operation, the feeding rate, applied voltage, and distance between the spinneret and collector are adjustable. Also, ambient factors, such as temperature, relative humidity, and airflow speed, deserve attention. When the above parameters are changed, the properties of electrospun nanofibers, such as surface morphology, diameter, tensile strength, etc., will change accordingly. For example, reducing the voltage may make the fiber thicker or even produce beads on the fiber, and increasing the humidity may create small holes on the fiber surface [31]. Herein, much attention should be paid to the effect of these parameters because they can facilitate optimization of the electrospinning process and obtain various nanofibers.

One shortcoming of nanofibers is their lack of mechanical integrity, making them unable to bear loads, which often limits their further application [33]. To solve this problem, the commonest method is to dope the fibers with fillers such as clay, nanotubes, nanoflowers, and metal particles [34]. Direct mixing of these fillers with an electrospinning solution and then electrospinning into nanofibers is the most accessible method. Nevertheless, these fillers are usually cytotoxic and not suitable for use in animal and human bodies. Many fiber post-treatment methods have been developed and adopted not only to enhance the tensile strength of fibers but also to avoid negative effects on organisms, including thermal treatment, uniaxial drawing, surface modification, etc. [34,35,36]. Thermal treatment melts the fibers and changes the pore size distribution to improve mechanical properties, while uniaxial drawing applies molecular orientation axially during the deformation phase to enhance fiber strength [37]. Surface modification mainly includes coating modification, cross-linking modification, and surface grafting reaction, which protect the fiber material from a harsh environment and reinforce the material’s performance [38,39,40]. Spinning parameters adjustments and fiber post-treating will change the structure of the fibers. Fibers with different structures have different porosities and specific surface areas, which can be adapted to different applications.

### 2.4. Diverse Biomaterials of Electrospinning

The materials generally used in electrospinning are polymers. Currently, over 200 polymers have been successfully spun into fibers through electrospinning [50]. Roughly, they are divided into natural polymers and synthetic polymers [51]. The commonly used polymers are summarized in Table 1, as well as their solvents. In most cases, synthetic polymers can be directly electrospun into nanofibers for applications, such as tissue engineering and drug delivery [52]. Electrospinning research mostly concentrates on polycaprolactone (PCL), poly(ethylene oxide) (PEO), polylactic acid (PLA), poly(D, L-lactide-co-glycolide) (PLGA), poly(vinyl alcohol) (PVA), and polyurethane (PU) due to their great formability and mechanical properties. Compared to synthetic polymers, natural polymers, including chitosan, dextran, alginate, hyaluronic acid, gelatin, chitin, etc., are more biocompatible and acceptable for usage in the human body. The majority of them are extracted from plants and animals [53]. However, since natural polymers have low solubility and most are polyelectrolytes, electrospinning with natural polymers is difficult to implement. As a result, natural polymers are frequently blended with specific synthetic polymers before being electrospun into fibers. 

### 2.5. Typical Structures of Electrospun Nanofibers in the Biomedical Field

#### 2.5.1. Core-Shell Fibers

With the remarkable development in the field of polymer and nanomaterial technology, there is an increasing amount of research related to core–shell nanostructure [65]. A great advantage of core–shell fibers (Figure 4a) is their ability to maintain the bioactivity of biomolecules like drugs and protein, making them favorable for situations that need special requirements for material degradation and release rates. Therefore, this kind of fiber is widely applied in tissue engineering and drug delivery [66]. 

As previously mentioned, because of its low cost and simple setup, coaxial electrospinning has made big progress in core–shell fiber production. Rastegar et al. prepared a platelet-rich fibrin (PRF)-loaded polycaprolactone/chitosan (PCL/CS-PRF) nanofibrous scaffold with a core–shell structure by coaxial electrospinning [67]. After testing, the addition of PRF core resulted in improved degradation rate, porosity, and wettability of the fibers. The excellent mechanical properties and osteoconductivity confirm that the core–shell fibers scaffold has tremendous potential for bone tissue engineering application.

Besides, emulsion electrospinning is another approach to fabricating core–shell fibers. It takes advantage of a water-in-oil (W/O) or an oil-in-water (O/W) emulsion by a conventional single needle for electrospinning [68]. The rapid evaporation of the oil phase leads to the viscosity of itself, making the biomolecule-containing aqueous droplets move to the center of the jet together [66]. After stretching and evaporation, the fibers with a special core–shell structure are formed on the collector. This electrospinning method allows the drug to be encased in a shell and released sustainably [69]. For example, as Zhan et al. reported, they successfully use emulsion electrospinning to obtain poly(vinyl alcohol) (PVA)/poly(acrylic acid) (PAA) fibers wrapping tangeretin (TAN). The result indicates that the PVA/PAA/Tan core–shell fibers have more durable release profiles and less possibility of releasing suddenly, making emulsion electrospinning a promising technique in the drug delivery field [70]. In addition, hyaluronic acid has been mixed with keratin and loaded into the core of poly(є-caprolactone)/polyethylene oxide by emulsion electrospinning and coaxial electrospinning separately [71]. The core–shell fibers demonstrate their ability to preserve bioactivity when used as a wound dressing. All signs indicate that emulsion electrospinning is a fabulous substitute for coaxial electrospinning. Nevertheless, the choice of surfactant and particle charge is so difficult that it hinders further development.

#### 2.5.2. Ribbon Fibers

Ribbon fibers (Figure 4f) have a flat cross-section. Compared with other electrospun nanofibers, ribbon fibers are quite difficult to fabricate. The production of such fibers requires careful design of various parameters, such as conductivity, solvent evaporation, and applied voltage, during the electrospinning process [72]. 

The most commonly used method for generating ribbon fibers is to utilize solvent evaporation. Fan et al. used a metastable zein protein solution in a solvent mixture to generate ribbon fibers [73]. At the initial stages, the internal fibers will remain in solution while the acetone evaporates. Due to vaporization of the internal solution, the fibers on the surface are compressed by air into a ribbon shape. According to this study, compared with a commercial air filter, the fiber mat with a ribbon structure has a better ability to capture sub-micron particulate pollutants and can reduce airflow resistance by clumping like cotton candy. Related studies have shown that the addition of gelatin causes rapid evaporation of the solution from the fiber matrix, resulting in the formation of ribbon fibers [74]. Pryadko et al. synthesized electrospun PHB/gelatin/Fe_3_O_4_ nanofibers as scaffolds based on previous studies [75]. This fiber with a flat structure has low crystallinity, suitable saturation magnetization, and is non-toxic to cells, which has great prospects for wound dressing, tissue engineering, and drug delivery. 

#### 2.5.3. Porous Fibers

The porosity of electrospun fibers usually refers to the interstices between the interconnected fibers. Porous fiber (Figure 4c) is a type of fiber that also shows porosity on the fiber surface or in the fiber matrix [76]. The method of generating porous fibers by a one-step process was first reported in 2001 [77]. In this study, three shapes of pores on the fiber surface were shown, including approximately ellipsoidal pores, regular circular pores, and ellipsoidal pits. 

The porous structure is usually the result of phase separation or ‘breath figures’. The phase separation method is induced to make the solution thermodynamically unstable. The fiber matrix is formed by the phase containing more polymer, while the pores are formed by the phase containing less polymer [76]. The ‘breath figures’ method is achieved through the regulation of humidity: water molecules in the air condense on the fiber surface due to the decrease in temperature caused by the evaporation of the spinning solvent, leaving imprints and forming pores [78]. Porous poly(lactic acid) (PLA) nanofibers have high biocompatibility, exhibiting prospects in the biomedical field [79]. Huang et al. employed both methods mentioned previously to prepare this fiber, giving some useful insight into the preparation of porous fibers [80].

One application of porous fibers is to take advantage of their larger specific areas. This feature can enhance their access to certain materials like photocatalyst. Porous poly(vinylidene fluoride) (PVDF) fiber film mats have been proven to anchor TiO_2_ well, which prevents complex energy-intensive separation processes and can be reused in water treatment. Additionally, because some air is trapped in the pores, porous fibers have better surface hydrophobicity to slow down the drug release rate. For instance, in the study of Chen et al., porous cellulose acetate (CA) fibers embedding thymol (THY) were fabricated. Compared with nonporous fibers, porous CA fibers extend the release time and have better antibacterial properties. There is no doubt that porous fibers show much-improved performance in many fields, and it is hoped that there will be lower cost and simpler manufacturing methods for their production.

#### 2.5.4. Beaded Fibers

Beaded fibers (Figure 4d) are often considered to be defective fibers. Adjustment of many parameters can lead to the jet fragmentation of dilute polymer solutions, creating beads significantly larger than the fiber diameter [81]. Several researchers have reported the effects of various electrospinning parameters on the bead size and shape. For example, Korycka et al. investigated the influence of voltage, solution flow rate, and solution viscosity on the polyvinylpyrrolidone (PVP) bead size [82].

Bead attachment increases the specific surface area and roughness of fibers. Because of these characteristics, beaded fibers can obtain low-pressure drop by packing density optimization, which has great utilization in the filtration industry [83]. Han et al. dissolved polylactic acid (PLA) with a relatively green ethyl acetate/N, N-dimethylformamide (EA/DMF) mixture solvent to fabricate environmentally friendly and degradable PLA beaded nanofibers through electrospinning [44]. As a result of the study, the beaded nanofibers were able to remove more than 98% of aerosol particles with an exceptionally low-pressure drop of 193.3 Pa. After actual tests, compared with ordinary commercial masks, the PLA beaded nanofiber membrane had higher filtration efficiency for pollutants PM2.5 and PM10 in a real haze environment, reaching 92.3% and 95.4%, respectively, making the PM index value relatively safe. In the pursuit of higher filtration performance, researchers have recently turned their attention to bilayer-beaded nanofibers. Kadam et al. reported a bilayer, beaded electrospun nanofiber membrane [83]. They prepared beaded fibers at a polyacrylonitrile (PAN) solution concentration of 4% *w*/*w*. The bead-free fibers were deposited on the beaded fibers by directly replacing the PAN solution with a concentration of 9% *w*/*w* after a period of electrospinning, without a time interval in between. Finally, bilayer fibers were formed. The changes in fiber thickness, density, and pore size caused by the beads improved the filtration capacity of the membrane. The filtration efficiency of these fibers reached 95.7% with a pressure drop of only 112 Pa. Aerosol contaminants of 0.3–5 μm can be captured.

In addition to changing electrospinning parameters, a special electrospinning setup can also be used to obtain beaded fibers [84,85]. In the study of Li et al., beaded fibers were made of polyvinylpyrrolidone K90-methylene blue (PVP K90-MB) and ethyl cellulose-ketoprofen (EC-KET) by a home-made eccentric spinneret [86]. This method allowed all components to be amorphous. The dual drug-controlled release profile provided by PVP-MB//EC-KET beaded fibers was demonstrated by in vitro dissolution tests, which opens new paths for combination therapy.

## 3. Recent Advances in Electrospun Nanofibers for Biosensors and Other Applications

### 3.1. Biosensors

A biosensor is a technological product combining biotechnology and electronic detection, usually consisting of a substance recognition element and a transducer [87]. The transducer is responsible for the sensitivity of biosensors. High sensitivity means that biosensors can successfully detect biological substances with weak signals from the biological recognition interacting with specific analytes. The biological recognition can be enzymes, antibodies, small molecules, genes, nucleic acids, and other active substances. The large specific surface area and small structure size of electrospun nanofibers equip themselves with more binding sites to recognize analytes and finally enhance the sensitivity and response speed [88]. Electrospun nanofibers generally play two roles in biosensor applications. Many polymer electrospun nanofibers have special properties that allow them to be used directly as induced functional layers for biosensors. Electrospun nanofibers can also be used as templates for the deposition of sensitive materials. Based on the sensing mechanism, biosensors include electrochemical biosensors, optical biosensors, impedimetric biosensors, thermometric biosensors, potentiometric biosensors, and pressure biosensors [10].

The principle of these sensors is mostly based on energy conversion. Electrochemical biosensors combine biomolecules and electrode surfaces to create a chemical reaction that converts biological signals into electrical signals. Optical biosensors convert optical signals into electrical signals according to the optical response involved in their sensing process or detection. Thermometric biosensors utilize the property of biological heat absorption and dissipation to induce temperature changes and convert the heat generated into a temperature change signal by means of a thermal energy converter. Potentiometric biosensors work on the principle of ion recognition to produce a change in point level or current. Pressure biosensors utilize the sensitivity of a piezoelectric material to the attached mass of a surface electrode to cause a change in vibration frequency to convert mechanical energy into electrical energy.

#### 3.1.1. Electrochemical Biosensors

Electrical characteristics are often sensed electrochemically in a biosensor to gather information from biological systems. The electrochemical component serves as the primary transduction factor in this process. While biosensor devices have used several types of recognition components, electrochemical detection techniques mostly rely on enzymes. This is often due to their distinct binding capabilities and biocatalytic functions. Electrochemical detection also employs other biorecognition components, including antibodies, nucleic acids, and micro-organisms.

Li et al. prepared size-controllable hollow nanofibers by coaxial electrospinning and subsequent calcination [89]. The large specific surface area of catalytic activity sites and the interface between heterogeneous structures greatly improved the electrocatalytic performance. The composite nanofibers (NFs) exhibited the highest electrocatalytic efficiency for glucose oxidation when the molar ratio of CuO to NiO was 0.4. They demonstrated a remarkable sensitivity of 1324.17 μA mM^−1^ cm^−2^ and a broad linear range spanning from 1 to 10,000 μM.

#### 3.1.2. Optical Biosensors

Electrospun membranes are composed of a network of interwoven fibers and holes that provide strong light scattering. At the macroscopic level, they often lack shine and are not transparent. These qualities may be used to create a coating that reduces glare or enhances the color of dyes included in the nanofibers. A wide range of optical detection techniques have been developed for sensing purposes, including the analysis of reflected, transmitted, scattered, or emitted light, specifically focusing on its intensity, spatial characteristics, and spectrum properties. The optical biosensors depend on either fluorescent signals (alterations in fluorescence intensity and/or spectrum features) or colorimetric signals (modifications in absorbance or reflectance characteristics). Electrospun nanofibers are efficient in expanding a particular region, hence enhancing the number of receptor molecules, enhancing sensitivity, and mitigating diffusion constraints.

Whispering gallery mode (WGM) resonators have garnered interest as optical biosensors because of their ability to quickly identify substances without the need for labeling [90]. Polyvinyl alcohol (PVA) bioreceptors containing M13 bacteriophage were prepared by near-field electrospinning [91]. Streptavidin-binding phage and rhodamine 6 G were used as representative virus-based biocapture agents and potent fluorescent sources, respectively. The resonator demonstrated selective binding of streptavidin, exhibiting a sensitivity of 0.008 nm/nM and a limit of detection (LoD) of 3 nM. This characteristic makes it a flexible sensing platform capable of detecting a wide variety of analytes.

#### 3.1.3. Thermometric Biosensors

Thermometric biosensors use the fundamental characteristic of biological responses, namely the absorption or release of heat. Thermometric biosensors are capable of detecting even small fluctuations in temperature. Conventional semiconductor-based thermistors or metal-based thermocouples have the disadvantages of high molding consumption and low flexibility. Thermometric biosensors composed of electrospun nanofibers, on the other hand, do not have these problems and can be monitored in real-time and non-contact.

Lee et al. fabricated a flexible temperature sensor, utilizing electrospun-aligned polyacrylonitrile (PAN)-based carbon nanofiber films [92]. This sensor demonstrates a high level of responsiveness to changes in temperature, particularly in the presence of various external stimuli. The temperature sensor, constructed using an aligned carbon nanofiber (ACNF) film, has exceptional sensitivity of 1.52% per degree Celsius (1C1), similar precision to thermometers available in the market, great linearity, a rapid reaction time of 1.2 s, and remarkable durability.

Some other examples of electrospun fibers used for biosensors are shown in Table 2.

As a systemic disease that induces changes in the immune system, cancer has become an important public health problem affecting the global population. According to the World Health Organization’s 2019 cancer report, cancer is the first or second cause of human death in 112 countries [101]. Constant efforts have been made to accurately diagnose cancer and cure cancerous patients, and electrospun nanofiber materials have also been applied here.

High sensitivity means that biosensors can successfully detect biological substances with weak signals from the biological recognition interacting with specific analytes [102]. The analytes can be cancer cells, exhaled gases, enzymes, circulating tumor cells, etc. That is, biosensors are also effective tools to diagnose cancer. The large specific surface area and small structure size of electrospun nanofibers equip themselves with more binding sites to recognize analytes and finally enhance the sensitivity and response speed to diagnose cancer [88].

**Figure 5:**
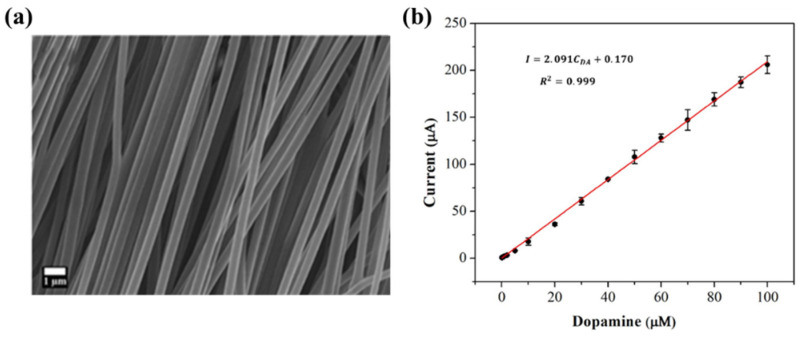
(**a**) SEM images of well-aligned NECNFs [104]. (**b**) Calibration curve of the peak current of the 4 h Co_3_O_4_ electrospun carbon nanofiber electrode with corresponding DA concentration [104].

Biosensors for cancer detection include gas sensors, immune sensors, gene sensors and electrochemical sensors [103]. Several recent electrospun fiber biosensors for detection of cancer analytes are shown in Table 3, including polymer materials and sensor types. Among them, electrochemical sensor, which produces chemical energy through the interaction between analyte and sensor elements to achieve real-time monitoring, is one of the most widely used biosensors in disease diagnosis. Electrochemical sensing offers higher chemical stability and lower cost compared to immunological methods. Yin et al. prepared nitrogen-doped carbon nanofibers by electrospinning (Figure 5a) and modified cobalt oxide nanograins (Co_3_O_4_) on them as electrochemical sensors for the detection of dopamine(DA) [104]. The ultra-high specific surface area of the electrospun carbon nanofibers not only facilitated the uniform dispersion of the nanoparticles but also promoted the electrochemical conductivity. DA levels in urine or blood can be used to detect rare and imperceptible neural crest tissue tumors. The electrochemical sensor showed high sensitivity and a good detection limit (9 nM) over a wide concentration range (0.01 to 100 μm), as shown in Figure 5b. Additionally, its excellent sensing properties and biocompatibility also suggest that this technology may provide rapid and safe detection of cancer.

### 3.2. Other Applications

#### 3.2.1. Tissue Engineering

Tissue engineering, also named regenerative medicine, is an emerging multidisciplinary area that replaced organ transplantation by using biologically active substances to repair or reconstruct damaged organs or tissues [113]. The key to this technology is the build-up of scaffolds. Tissue engineering scaffolds should mimic the properties of tissue extracellular matrix (EMC) as much as possible, taking into account cell–to–cell interactions [114,115]. Nanofiber systems have become a target for tissue engineering scaffold fabrication throughout the last decade [116]. Electrospun nanofiber products have a large specific surface area and excellent porosity, akin to natural EMC networks [117]. Also, the requirement for biocompatibility and degradation rate of scaffolds can be met by selecting appropriate electrospinning polymer materials. Herein, many researchers are concentrating their efforts on developing electrospinning applications in the tissue engineering field, including bone tissue, blood vessel tissue, skin tissue, etc. [118,119,120].

**Figure 6:**
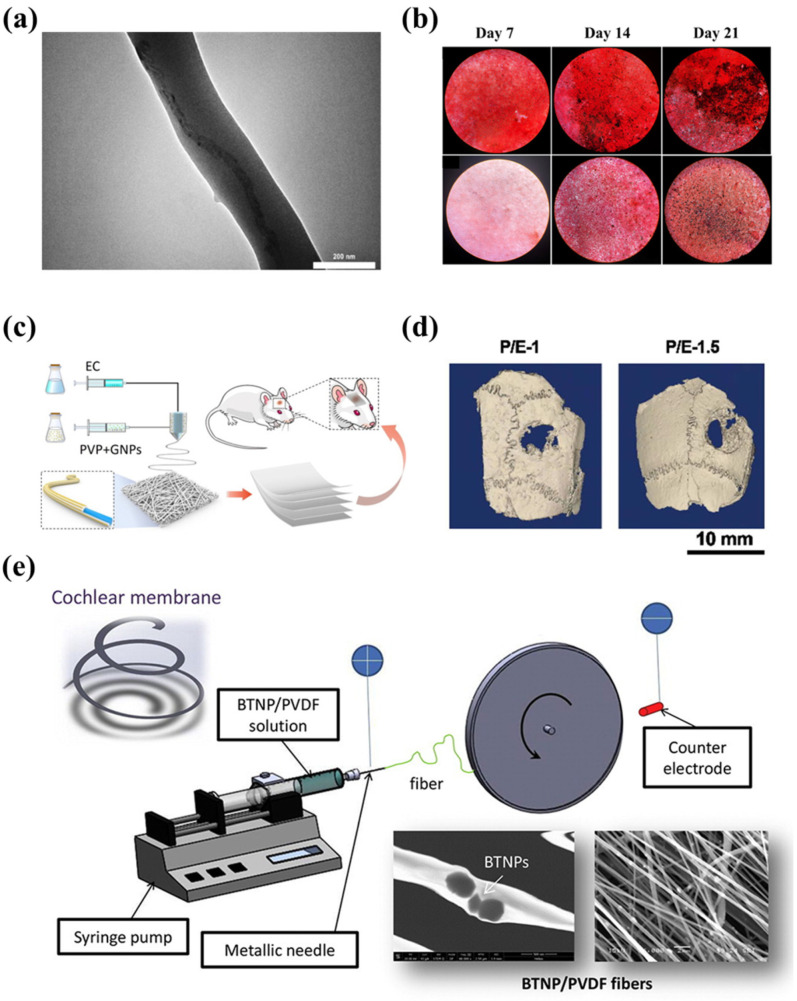
(**a**) TEM image of PHB-S-1% MWCNTs electrospun fiber [121]. (**b**) Inverted light microscopic images showing Alizarin red S straining of MG-63 cells on PHB-S-1%MWCNTs electrospun scaffolds and control samples at days 7, 14 and 21 [121]. (**c**) The GNPs-incorporated PVP/EC coaxial electrospun nanofiber scaffold used in bone tissue regeneration [123]. In vivo bone regeneration of the GNPs-loaded PVP/EC coaxial electrospun nanofiber scaffold: (**d**) the reconstructed 3D micro-CT images [123]. (**e**) An electrospinning device with its BTNP/PVDF product [126].

Asl et al. proposed a Polyhydroxybutyrate-starch(PHB-S)/MWCNTs nanotube electrospun nanocomposite as shown in Figure 6a [121]. The incorporation of MWCNTs further improved fiber surface roughness and hydrophilicity. Figure 6b shows the calcium phosphate production and deposit by MG63 cells by the alizarin red-S assay, which indicates that the PHB-S-1%MWCNTs electrospun scaffolds had the highest calcium deposition and were almost brown to black in color. This suggests that the PHB-S-1%MWCNTs may be a promising bone substitute for bone tissue regeneration. However, one issue to consider with electrospun scaffolds for bone tissue engineering is mechanical strength, as the modulus of polymer materials is lower than that of natural hard tissues [122]. Huang et al. encapsulated gold nanoparticles (GNPs) into polyvinylpyrrolidone/ethylcellulose (PVP/EC) nanofiber scaffolds by coaxial electrospinning and characterized the morphology and physicochemical properties of the scaffolds(Figure 6c) [123]. Compared with pure PVP/EC nanofibers, the introduction of GNPs gives the scaffold better mechanical properties and porosity, resulting in excellent osteogenic bioactivity, and the ability of the scaffold to accelerate bone regeneration was confirmed by implantation tests in the defective area of rat skull as displayed in Figure 6d.

The short-lived efficacy often limits the development of electrospun scaffolds. The ensuing fibrosis and chronic inflammation can lead to stent implantation failure; hence, researchers have begun applying electrospun materials with special properties to act as scaffolds. Piezoelectric polymers have received much attention as they are claimed to possess an electric charge that enhances cell adhesion and proliferation, which is highly beneficial for the implantation of scaffolds [124]. Polyvinylidene fluoride (PVDF) and its copolymers are common piezoelectric polymers [125]. Figure 6e shows a piezoelectric composite fiber of electrospun PVDF and its fabrication method [126]. In this study, Mota et al. added barium titanate nanoparticles (BTNP) to it and made aligned fibers improve the performance of PVDF fiber membranes by means of high-speed rotating discs. The obtained results showed that inner-ear epithelial cells and neural-like cells cultured on the fiber membranes showed better viability.

Electrospun nanofibers are ideal for tissue cell inoculation, adhesion, and proliferation due to their high porosity. The scaffold pore size, which plays an important role in nutrient and oxygen diffusion, as well as waste elimination, has a significant impact on the continued operation of transplanted cells [127]. To emulate natural EMC, increasing the porosity of electrospun scaffolds becomes a major challenge. One way is to add sacrificial fibers into scaffolds and then wash them out [128]. For example, poly(ethylene oxide) (PEO) is a water-soluble polymer commonly utilized as a sacrificial fiber material [129,130]. After washing them away with deionized water, insoluble fibers with large pores are obtained. Other methods include salt leaching, using liquid bath collectors, gas foaming, and so on [131]. However, attention should be paid to the fact that an increase in porosity often brings about the risk of mechanical strength weakening. Meanwhile, another potential problem that electrospun nanofibers for tissue engineering may encounter is the toxicity of the organic solvent used. These toxic organic solvents are harmful to living cells. Therefore, new technologies need to be developed to facilitate the clinical application of electrospun scaffolds.

#### 3.2.2. Drug Delivery

The goal of drug delivery devices is to maximize therapeutic effects and minimize unwanted side effects. The inherent flexibility, large specific surface area, and small diameter allow the electrospun nanofibers to exhibit superior loading capacity and encapsulation efficiency [132]. The use of electrospun nanofibers for drug delivery dates back as far as 2002: poly(lactic acid) (PLA), poly(ethylene-co-vinyl acetate) (PEVA), and the 50:50 blend of them were utilized to release tetracycline hydrochloride, manifesting a preferred drug-release profile over the drug carriers produced by a conventional casting technique [133]. To date, electrospun nanofibers have been explored to load many types of drugs, including antibiotics, proteins, ribonucleic acid (RNA), deoxyribonucleic acid (DNA), and living cells [134,135,136].

There are different approaches for drug loading in electrospun nanofibers, including blending, coaxial electrospinning, and post-processing. The drug-nanofiber interactions differ for different drug loading approaches, leading to different drug release kinetics. The properties of the drug, the characteristics of the polymer, and the fiber morphology all affect the release rate of the drug. Research has been dedicated to controlling the drug release rate. Wu et al. investigated the drug release behavior of poly(D, L-lactide-co-glycolide) (PLGA) mono-/bicomponent electrospun films and reported the mechanism of drug release based on blended electrospun fibers [137]. They found that there are three stages in the drug release profiles: stage 1 is influenced by fiber swelling and diffusion, stage 2 is influenced by film structure, and stage 3 is influenced by polymer degradation. It is possible to control the dose and duration of drug release based on this study.

**Figure 7:**
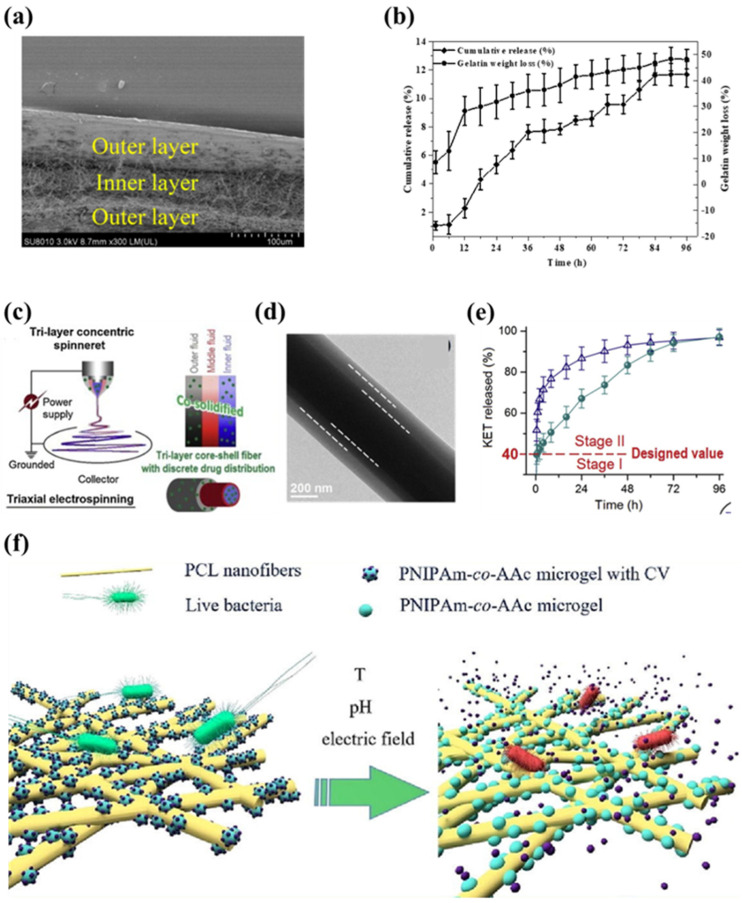
(**a**) The cross-section of the ethylcellulose/gelatin/ethylcellulose multilayer film [138]. (**b**) The curcumin release profiles and weight loss from the ethylcellulose/gelatin/ethylcellulose multilayer film [138]. (**c**) The modified triaxial electrospinning for preparing the CA/KET-CA-PVP/KET fibers [140]. (**d**) The TEM images of the CA/KET-CA-PVP/KET fibers [139]. (**e**) The dual-stage release performance of KET from the tri-layer core–shell nanofibers [139]. (**f**) The schematic diagram of poly(N-isopropylacrylamide-co-acrylic acid) (PNIPAm-co-AAc) microgel-loaded polycaprolactone (PCL) nanofibers temperature-, pH- and electro-responsive materials [145].

As described in Section 3.2.1, core–shell fibers were used to mitigate burst release. Multilayer electrospun films have a similar effect. In the work of Wang et al., sequential electrospinning was used to manufacture such films with an inner layer of curcumin-loaded gelatin nanofibers and outer layers of ethylcellulose nanofibers (Figure 7a) [138]. By virtue of hydrophobic outer layers, the water vapor permeability of the hydrophilic inner layer was reduced. The multilayer film can release curcumin continuously for 96 h and maintain its antioxidant activity, which controls the release very well (Figure 7b). As a further derivation of the core–shell structure, the triaxial fiber also opens up more possibilities for drug delivery. Yang et al. added bank cellulose acetate (CA) nanofibers between the Polyvinylpyrrolidone/ketoprofen (PVP/KET) outer fibers and the cellulose acetate/ketoprofen (CA/KET) core layer through improved triaxial electrospinning as shown in Figure 7c,d [139]. The bank CA nanofiber layer acted as a slow-release polymer matrix. The drug-discrete distribution of the triaxial nanofibers provided a better dual-stage KET release profile compared to conventional core–shell fibers, which is a good mechanism for adjusting the behavior of drug release (Figure 7e).

In recent years, smart or stimuli-responsive electrospun nanofibers that can respond to small environmental changes have received increasing attention in drug release control [140]. They can start or stop drug release depending on external stimuli, such as pH, light, magnetic fields, electric fields, etc. [141,142,143,144]. An example is a study by Khrystonko et al. on the preparation of poly(N-isopropylacrylamide-co-acrylic acid) (PNIPAm-co-AAc) microgel-loaded polycaprolactone (PCL) (Figure 7f) [145]. The prepared nanofibers were loaded with crystal violet (CV) as model drugs. The amount of released drug could be effectively regulated according to the magnitude of the applied pulse voltage. Moreover, long-term temperature and pH corresponding release were also demonstrated in in vitro drug testing. So far, it has been verified that this nanofiber had temperature-, pH- and electro-response. The application of smart nanofibers enables further control of drug release. They have shown their potential in drug delivery, especially for targeted therapies, and gradually becoming a trend. Currently, electrospun fibers for drug transport still face the problem of toxic solvent residues, which may be released with the drug and have adverse effects. Therefore, the need to choose green solvents such as water or the application of new technologies such as melt electrospinning are becoming increasingly critical.

## 4. Conclusions

With the interdisciplinary development of nanotechnology, nanomaterials are becoming more functional and practical, thereby playing an increasingly important role in biomedical fields. Among them, nanofibers are notable for their biomimetic property. Electrospinning is a low-cost and facile method for preparing nanofibers. Nanofiber-based biosensors provide many advantages compared to conventional biosensors, such as enhanced reactivity, heightened sensitivity, wider detection range, and cost-efficiency. This review covers the basic principles of electrospinning, the typical electrospun nanofiber structure utilized in the field of biomedicine, and the applications of electrospinning for biosensors, and other biomedical fields.

Although electrospinning technology has proven its advantages in the biomedical field, it still faces many challenges. An efficient mechanism for the mass production of electrospun fibers is urgently needed. Long fabrication times and low yields have hindered the commercialization of electrospinning. The mass production approaches presented in the article, including needleless electrospinning and multi-needle electrospinning, need to be further improved if they are to be implemented industrially. Electrospinning devices with high production rates usually face problems of poor free liquid stability and difficult control of multiple jets. Xiong et al. constructed a high curvature annular pre-Taylor cone on the free liquid surface to form multiple jets. They used a mushroom spinneret to ensure consistent motion of multiple jets, showing an excellent productivity of 13.7 g/h [146].

Because electrospun nanofibers usually work by implantation into or direct contact with the human body, another issue that needs attention is material safety. There are only a few clinical trials on electrospun fiber products, not to mention approval by the Food and Drug Administration (FDA) and European Medicines Agency (EMA) is still needed. Although synthetic polymers such as PVA are approved by the FDA for clinical use, their bioactivity and biocompatibility are relatively lacking. One solution is to combine natural polymers with synthetic polymers, taking advantage of both the physical properties of the synthetic polymers and the bioactivity of the natural polymers [147]. The development of a non-toxic, biocompatible, electrospun material that degrades in the human body remains a great challenge. Due to the vastly different metabolic systems, it is not enough to test the effects of electrospun fiber biomedical products on small animals such as rats. More clinical trials need to be conducted to improve the reproducibility of the experiments and achieve practical applications. In summary, electrospinning is a rapidly growing and promising technology in the biomedical field.

## Figures and Tables

**Table 1:** The commonly used polymers of electrospinning for biomedicine.

	Polymer	Solvent	Fiber Diameter	Structure	Biomedical Application	Refs.
Natural polymers	Chitosan/PEO	Acetic acid/dimethyl sulfoxide (10:1 *w*/*w*)	181–395 nm	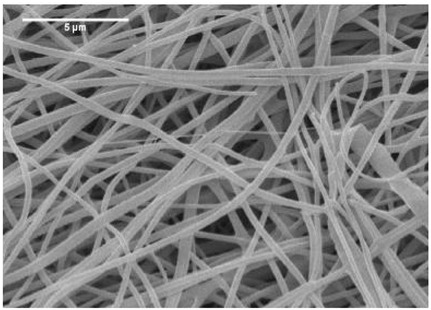	Periodontal regeneration	[54]
	Dextran	Boric acid	550–600 nm	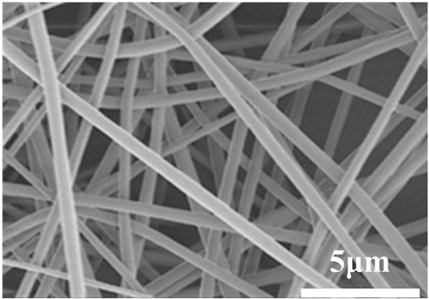	Drug delivery	[55]
	Alginate/PEO	Water	109–161 nm	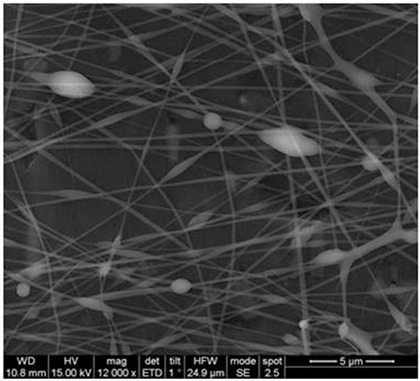	Drug delivery	[56]
	Hyaluronic acid	Na4OH/N,N-Dimethylformamide (DMF) (4:1 *w*/*w*)	27–51 nm	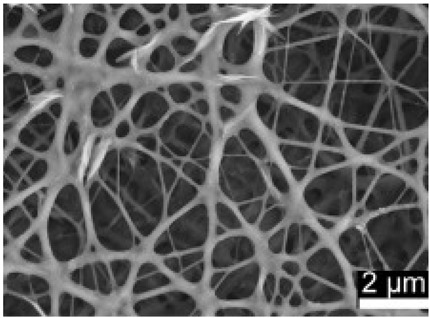	Ophthalmology; drug delivery; medical implants	[57]
	Gelatin	Detect 17α- Water	400–1000 nm	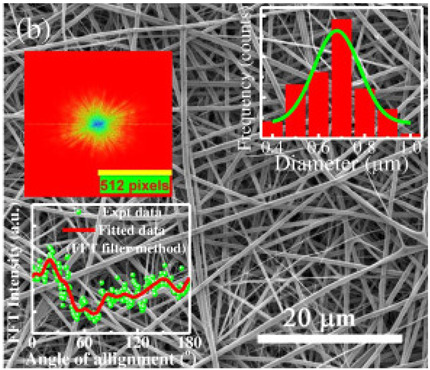	Biosensor	[58]
Synthetic polymers	PCL	DMF/dichloromethane (DCM) (1:1 *w*/*w*)	236–332 nm	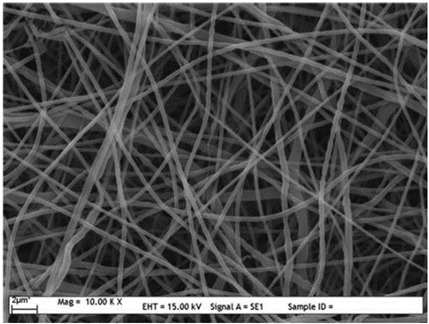	Tissue engineering	[59]
	PCL/PEO	DMF/Chloroform (1:9 *w*/*w*)	541–753 nm	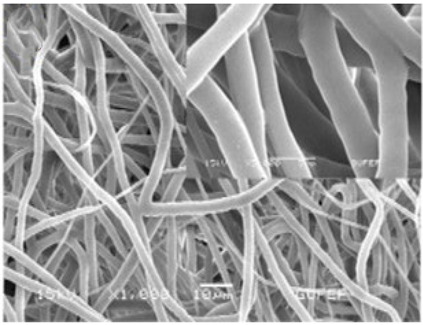	Drug delivery	[60]
	PLA	Chloroform/dimethyl sulfoxide (DMSO) (75:25 *w*/*w*)	232.4–498.3 nm	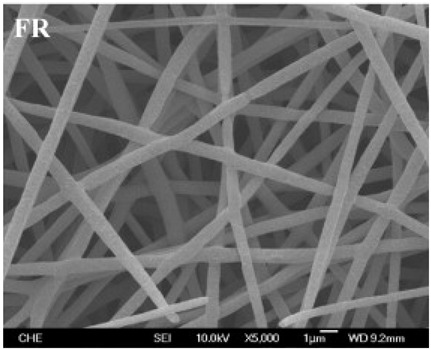	Tissue engineering	[61]
	PLGA	Tetrahydrofuran (THF)/DMF (3:1 *w*/*w*)	506–802 nm	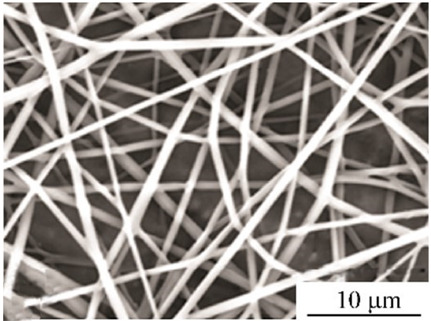	Drug delivery	[62]
	PVA	Phosphate buffer	187–282 nm	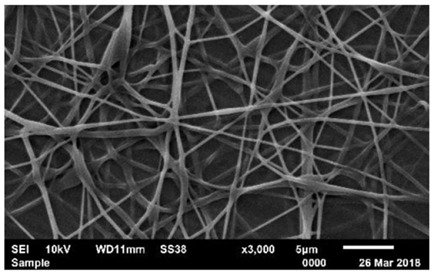	Enzyme immobilization	[63]
	PU	DMF	580–900 nm	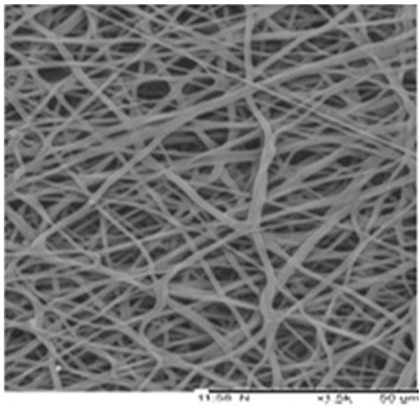	Wound dressing	[64]

**Table 2:** Electrospinning for biosensors.

Polymer	Solvent	Fiber Diameter	Structure	Refs.
C-phycocyanin/PVA	water	150–200 nm	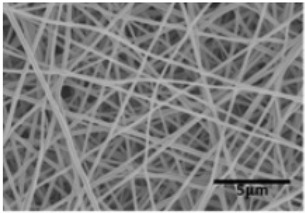	[93]
Aminoacylase/PVA	water	290–310	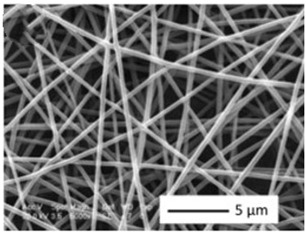	[94]
PVDF-PEI/Anti-METH	DMF/Ac (2:8; *v*/*v*)	382–408 nm	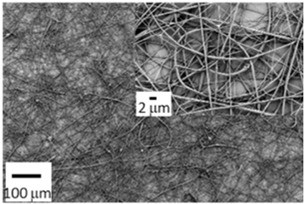	[95]
PAN	DMF		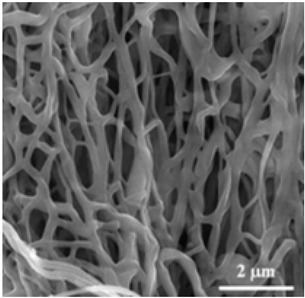	[96]
PAN	DMF	657–697 nm	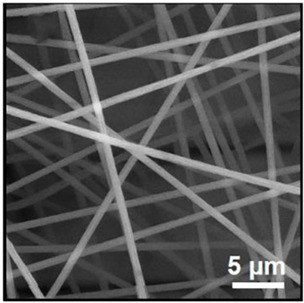	[97]
PAA/PVA	water	290–390 nm	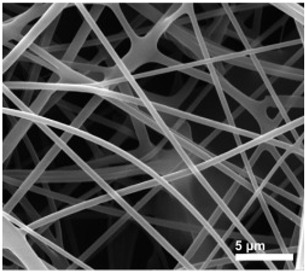	[98]
CoAc/PAN	DMF		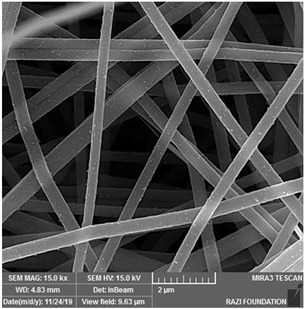	[99]
β-caryophyllene/PCL	Chloroform/acetone (1:1 wt)	500–900 nm	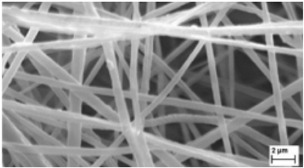	[100]

**Table 3:** Recent research on electrospun nanofibers for cancer diagnosis.

Polymer Matrix	Sensor Type	Analytes	Cancer Diagnosed	Detection Method	Ref.
Polyamide 6 (PA6) and poly(allylamine) (PAH)	Immunosensor	Cancer antigen (CA19-9)	pancreatic cancer	Impedance spectroscopy	[105]
Polyvinylpyrrolidone (PVP)	Gas sensor	Ammonia, ethanol isoprene, acetaldehyde, isoprene and acetone	Lung cancer	Electrochemical	[106]
Poly(vinyl alcohol) (PVA)	Electrochemical immunosensor	Carcinomaembryonic antigen (CEA)	/	Electrochemical	[107]
Polyvinyl pyrrolidone (PVP)	Electrochemical sensor	Hydrogen peroxide (H_2_O_2_)	Breast cancer	Electrochemical	[108]
Polyacrylonitrile (PAN)	Fluorescent sensors	Cancer cells	Liver cancer	Molecular imprint; Enzyme-free signal amplification	[109]
Polyaniline	Electrochemical sensor	Cyclooxygenase-2 (COX-2)	/	Electrochemical	[110]
Polyacrylonitrile (PAN)	Immunosensor	Epidermal growth factor receptor (EGFR or ErbB2)	Breast cancer	Impedance spectroscopy	[111]
Polyacrylonitrile (PAN)	Fluorescent sensors	MicroRNA-21 (Mir-21)	Cholangiocarcinoma	Fluorescence	[112]

## Data Availability

Not applicable.
